# Characterization of Expressed Sequence Tags From a *Gallus gallus* Pineal Gland cDNA Library

**DOI:** 10.1002/cfg.484

**Published:** 2005

**Authors:** Stefanie Hartman, Greg Touchton, Jessica Wynn, Tuoyu Geng, Nelson W. Chong, Ed Smith

**Affiliations:** 1 Comparative Genomics Lab Department of Animal and Poultry Sciences Virginia Tech Blacksburg VA 24061 USA; 2 Division of Cardiology Department of Medicine University of Leicester Leicester UK; 3 2250 Litton Reaves Hall Blacksburg VA 24061 USA

## Abstract

The pineal gland is the circadian oscillator in the chicken, regulating diverse functions ranging from egg laying to feeding. Here, we describe the isolation and characterization of expressed sequence tags (ESTs) isolated from a chicken pineal gland cDNA library. A total of 192 unique sequences were analysed and submitted to GenBank; 6% of the ESTs matched neither GenBank cDNA sequences nor the newly assembled chicken genomic DNA sequence, three ESTs aligned with sequences designated to be on the Z_random, while one matched a W chromosome sequence and could be useful in cataloguing functionally important genes on this sex chromosome. Additionally, single nucleotide polymorphisms (SNPs) were identified and validated in 10 ESTs that showed 98% or higher sequence similarity to known chicken genes. Here, we have described resources that may be useful in comparative and functional genomic analysis of genes expressed in an important organ, the pineal gland, in a model and agriculturally important organism.


**Comparative and Functional Genomics**
*Comp Funct Genom* 2005; **6**: 301–306.
Published online 11 July 2005 in Wiley InterScience (www.interscience.wiley.com). **DOI:** 10.1002/cfg.484

**Research Article**

### Characterization of expressed sequence tags from a *Gallus gallus* pineal gland cDNA library

Stefanie Hartman[1], Greg Touchton[1], Jessica Wynn[1], Tuoyu Geng[1], Nelson W. Chong[2] and Ed Smith[1]*
[1] *Comparative Genomics Lab, Department of Animal and Poultry Sciences, Virginia Tech, Blacksburg, VA 24061, USA*
[2] *Division of Cardiology, Department of Medicine, University of Leicester, Leicester, UK*

**Correspondence to:*
*Ed Smith, 2250 Litton Reaves Hall, Blacksburg, VA 24061, USA.*
*E-mail: esmith@vt.edu*

Received: 12 January 2005
Revised: 22 May 2005
Accepted: 1 June 2005

### Abstract

**The pineal gland is the circadian oscillator in the chicken, regulating diverse functions ranging from egg laying to feeding. Here, we describe the isolation and characterization of expressed sequence tags (ESTs) isolated from a chicken pineal gland cDNA library. A total of 192 unique sequences were analysed and submitted to GenBank; 6% of the ESTs matched neither GenBank cDNA sequences nor the newly assembled chicken genomic DNA sequence, three ESTs aligned with sequences designated to be on the Z_random, while one matched a W chromosome sequence and could be useful in cataloguing functionally important genes on this sex chromosome. Additionally, single nucleotide polymorphisms (SNPs) were identified and validated in 10 ESTs that showed 98% or higher sequence similarity to known chicken genes. Here, we have described resources that may be useful in comparative and functional genomic analysis of genes expressed in an important organ, the pineal gland, in a model and agriculturally important organism. Copyright © 2005 John Wiley & Sons, Ltd.**

**Keywords:** **Gallus gallus; pineal gland ESTs, SNPs**

### Introduction

Circadian rhythm is a general characteristic of living organisms. Both physiological and genetic factors involved in this process continue to be very widely investigated in different organisms. In mammalian and avian systems, it is a general consensus that the physiological and genetic processes of biological rhythms occur in a loop. The molecular mechanisms that control the loop appear to be conserved among diverse species. The avian circadian rhythm is unique as it involves multiple organs whose inputs and interactions influence the oscillatory patterns of rhythmic behaviour (Ebihara *et al.*, 1987). Some of the positive and negative regulator genes involved in the autoregulatory feedback loop mechanism for the circadian oscillator in the pineal gland have been described in diverse birds, including the quail (Yoshimura *et al.*, 2000) and chicken (Okano *et al.*, 2001).

The chicken pineal gland is an important model for vertebrate circadian clock systems because of its ability to retain circadian rhythm in culture. Several important genes have been identified in the pineal gland. One important component of the autoregulatory feedback loop of the circadian oscillator is the negative regulator gene, *cPer2*; the gene products of *cBmal1, cBmal2* and *cClock* form heterodimers that bind to a promoter sequence of *cPer2* and activate transcription (Okano *et al.*, 2001). The photoreceptor pinopsin has been shown to be present, although its expression responds exclusively to light and not circadian patterns. The arylalkylamine *N*-acetyltransferase (AA-NAT) gene product, however, has been directly linked to melatonin production in a circadian rhythm (Takanaka *et al.*, 1988). In addition, GCAP1, GCAP2 and GC, genes that are important in resetting rods and cones after light exposure, have been identified in the pineal gland (Semple-Rowland, 1999).

Copyright © 2005 John Wiley & Sons, Ltd.

Since the chicken is considered an excellent model for further understanding the genetic and molecular basis of rhythmic behaviour, here we investigated the characteristics of expressed sequence tags (ESTs) isolated from the chicken pineal gland. While previous work by Hubbard *et al.* (2005) has yielded a number of ESTs in such important functional tissues as the liver, pancreas, heart, cerebellum, kidney and ovary, none has been described to date from the pineal gland. Bailey *et al.* (2003) used microarray technology to evaluate pineal genes expressed in periods of light and darkness with a focus on function rather than sequence comparisons. The primary goal of our study was to identify novel genes that could be useful in comparative genome analysis of the molecular mechanisms that underlie rhythmic behaviour. Additionally, we evaluated the level of variation in selected ESTs that matched known chicken genes using *in silico* analysis followed by PCR-based resequencing for validation.

### Materials and methods

### Sequence analysis

The ESTs were obtained from a previously described chicken pineal gland-cDNA library (Chong *et al.*, 2000). Briefly, the library was established from 10–11 day-old White Leghorn birds under 12 h light. The ESTs were produced from single-pass sequencing of randomly selected clones, processed by a modification of the toothpick PCR described by Smith *et al.* (2001). The modification involved first converting the original library from HybriZAP2.1 into phagemid, using the manufacturer's (Stratagene, La Jolla, CA92037) recommendation. The ESTs were characterized using BLAT (**http://genome.ucsc.edu/cgi-bin/hgBlat?command=start&org=Chicken&db=galGal2&hgsid=30295885**) and BLAST to identify database matches corresponding to the recently released chicken genomic DNA sequence and known genes in GenBank, respectively.

The chicken radiation hybrid panel (Morisson *et al.*, 2002) was used to map VTEST71 in order to validate the *in silico* chromosomal location of the EST. Forward and reverse primers specific for the EST, designed using Primer 3 (Rozen and Skaletsky, 1997), were used for the genotyping. The forward and reverse primers were 5′-GAT TTC AAA ACG GAC TTG AG-3′ and 5′-TGA GCA GTC ACT TTT AGC ATT-3′, respectively. The PCR was carried out in a final volume of 10 µl containing 1.5 mM $Mg^{2+}$ Buffer (Eppendorf, Westbury, NY), 200 µM dNTPs, 70 µg primer (MWG Biotech), 1 U Taq (Eppendorf), and 5 ng template. The cycling was performed using a Mastercycler (Brinkmann, Westbury, NY) with the following program: initial denaturation at 95 °C for 5 min followed by 95 °C for 45 s, 55 °C for 45 s, 72 °C for 45 s for a total of 38 cycles of denaturation, annealing and extension, respectively. A final extension at 72 °C was carried out for 7 min. The PCR product was run on a 2% agarose gel stained with ethidium bromide, and scored as 0, 1 or 2 for absent, present, or ambiguous, respectively. Mapping results were determined by the Morisson lab from these data.

### SNP analysis

An *in silico* analysis of 10 ESTs that closely matched chicken genes was used to identify candidate SNPs in the ESTs according to the pipeline protocol of Buetow *et al.* (1999). Validation of the candidate SNPs for three of the ESTs was carried out by PCR-based resequencing of amplicons from 10 unrelated commercial birds, using previously described protocols (Smith *et al.*, 2001).

### Results and discussion

Of the 200 clones sequenced, a total of 192 sequences exceeded a Phred quality score of 30 (Ewing *et al.*, 1998). These 192 sequences were submitted to GenBank and have been assigned accession numbers (Table 1; and at **http://filebox.vt.edu/users/esmith/Hartman_Va_Tech_CFG_supplement/Hartman_Va_Tech_Table 1.doc**). A total of 17 ESTs (9%) matched neither GenBank cDNA sequences nor the newly assembled chicken genomic DNA sequence. Additionally, only 80 ESTs matched known chicken gene or cDNA sequences. All but 28 ESTs aligned with genomic DNA sequences assigned to chicken chromosomes. Ninety-one (about 47%) ESTs aligned with sequences assigned to macrochromosomes (GGA) 1–6, and four sequences aligned to genomic DNA sequences assigned to the Z chromosome. An additional three ESTs aligned with sequences designated to be on the Z_random, while one matched

Copyright © 2005 John Wiley & Sons, Ltd. 

**Table 1.** Characteristics of the DNA sequences of cDNA clones randomly selected from a chicken pineal gland cDNA library*†

| EST-ID | SIZE (bp) | dbEST_Id | GenBank Acc. No. | $Ovl_{start}$ | $Ovl_{end}$ | Homo (%) | CHRO | SeqStart | SeqEnd | Identity |
|---|---|---|---|---|---|---|---|---|---|---|
| VTEST1 | 659 | 21 998 612 | CK983005 | 14 | 644 | 98.20 | Un | 97 081 430 | 97 082 805 | *Onchorhynchus mykiss* proteasome subunit/AF361366/80/311/387 |
| VTEST2 | 648 | 22 000 954 | CK985347 | 5 | 648 | 98.00 | 7 | 27 240 166 | 27 240 803 | None |
| VTEST3 | 1152 | 22 000 965 | CK985358 | 439 | 692 | 91.60 | Un | 119 005 139 | 119 005 611 | None |
| VTEST4 | 658 | 22 000 976 | CK985369 | 32 | 658 | 99.70 | 2 | 22 613 997 | 22 614 623 | None |
| VTEST5 | 838 | 22 000 987 | CK985380 | 163 | 583 | 93.00 | 1 | 96 381 892 | 96 399 994 | *Gallus gallus* amyloid precursor protein mRNA/AF042098.1/95/207/217 |
| VTEST6 | 579 | 22 000 998 | CK985391 | 162 | 483 | 95.00 | Un | 59 988 455 | 59 988 845 | None |
| VTEST6 | 579 | | | 162 | 445 | 97.40 | 1 | 181 404 458 | 181 404 473 | None |
| VTEST7 | 674 | 22 001 009 | CK985402 | 18 | 674 | 97.80 | 1 | 116 212 103 | 116 216 443 | *Mus musculus* trafficking protein particle complex 2/BC034845.1/84/355/422 |
| VTEST8 | 892 | 22 001 020 | CK985413 | | | | | | | *Gallus gallus* gene for Bcl-2 protein/D11382.1/84/219/259, *Gallus domesticus* mRNA for PCKBCL2/Z11 961.1/83/216/259 |
| VTEST9 | 678 | 22 001 031 | CK985424 | 6 | 632 | 99.40 | 2 | 105 326 626 | 105 334 057 | *Gallus domesticus* mRNA for transthyretin/X60 471.1/99/622/626, *Gallus gallus* finished cDNA/BX935 181.1/100/279/279 |
| VTEST10 | 693 | 21 998 613 | CK983006 | 51 | 693 | 99.70 | M | 9131 | 9773 | *Gallus gallus* mitochondrial DNA/AP003318.1/100/643/643 |
| VTEST11 | 686 | 21 998 624 | CK983017 | 32 | 593 | 96.50 | Un | 59 912 274 | 60 850 735 | None |
| VTEST12 | 685 | 21 998 635 | CK983028 | 54 | 684 | 97.00 | Un | 63 086 860 | 63 087 492 | *Gallus gallus* chromosome UNK clone TAM33-40B24/AC140947.1/90/481/533, |
| VTEST12 | 685 | | | 54 | 685 | 97.00 | 1 | 161 372 482 | 161 373 115 | *Gallus gallus* clone WAG-71G10/AC091751.2/85/484/566 |
| VTEST13 | 679 | 21 998 646 | CK983039 | 87 | 674 | 89.30 | 15 | 7 488 642 | 7 500 400 | *Danio rerio* similar to phosphotidylinositol transfer protein/BC047829.1/88/66/75 |
| VTEST14 | 640 | 22 000 895 | CK985288 | 212 | 576 | 99.20 | Un | 150 293 259 | 150 294 025 | *Gallus gallus* finished cDNA/BX932 334.1/99/596/600 |
| VTEST15 | 675 | 22 000 906 | CK985299 | | | | | | | *Gallus gallus* finished cDNA/BX935 387.1/84/420/500, *Gallus gallus* mRNA for transrationally controlled tumour protein/D26312.1/83/416/500 |
| VTEST16 | 644 | 22 000 917 | CK985310 | 15 | 644 | 99.70 | 1 | 47 401 719 | 47 404 831 | *Bos taurus* mRNA for ribosomal protein L3/Z29 555.2/84/522/620, *B. taurus* ribosomal protein L3 (Rpl3)/NM_174 715.1/84/522/620 |

* Where $Ovl_{start}$ and $Ovl_{end}$ are the start and end positions in the query sequence that overlaps the assembled chicken genomic DNA sequence; Homo (%), the percentage similarity of the query sequence to the assembled chicken DNA sequence in the matched region; CHRO, the chromosome to which the matched sequence is assigned by BLAT which are as defined (6); SeqStart and SeqEnd, the positions of the assembled chicken DNA sequence at which the matched EST sequence or the query starts and ends, respectively; GenBank matches of the respective EST: each description of the database sequence is followed by the Accession No., the % identity and the overlap, showing the lengths of the query and database sequences in the overlap. A detailed Table 1 is available at **http://filebox.vt.edu/users/esmith/Hartman_Va_Tech_CFG_supplement/Hartman_Va_Tech_Table 1.doc**

Copyright © 2005 John Wiley & Sons, Ltd.

a W chromosome sequence. The highest number of ESTs, 30, matched sequences from chromosome 1, while none aligned with sequences from microchromosomes 16, 19, 21, 25 and 26. Nineteen ESTs (11.7%) aligned with sequences designated 'unknown,' which are reported by the International Chicken Genome Sequencing Consortium (2004) to be about 12% of the chicken genome. Seven ESTs matched sequences assigned either to more than one region of a chromosome or on different chromosomes. A few ESTs aligned with sequences from *Escherichia coli*, which could be due to bacterial contamination or simply to conserved sequences. The chromosomal assignments of some of the ESTs should be considered putative, as there are still many errors in the draft chicken genomic DNA sequence. The incompleteness of the *Gallus gallus* DNA sequence may also account for the relatively high percentage of ESTs that showed no significant sequence similarity to known chicken sequences.

The chromosomal assignment of VTEST71 to chromosome 18, based on the sequence alignment with the recently released genomic DNA, was confirmed by radiation hybrid mapping. VTEST71 is designated as locus VTC08 on the chicken radiation hybrid map and is flanked by MCW0217 and ADL0290, with LOD scores of 10.7 and 13.1, respectively. VTEST71 showed 99% sequence similarity to chicken histone protein H3 and 95% identity with human Histone H3.3 (AK130772). Previously, chicken H3 was also mapped to chromosome 18 by RFLP, while the human H3 was linked to chromosome 17 (Levin *et al.*, 1994).

A total of 22 SNPs were identified and validated in the three ESTs scanned (data not presented). Eight of the SNPs were non-synonymous and are described in Table 2. All the SNPs appear to be novel and have not been previously described (Smith *et al.*, 2002; Wong *et al.*, 2004). Therefore, these SNPs, although few, may be useful in efforts to assign phenotypes to genotypes and identifying the effects of the three genes on different chicken traits, e.g. knowledge of the function of cofilin, an essential protein for depolymerization of actin filaments, is still limited (Arber *et al.*, 1998).

**Table 2.** Sequence contexts of SNPs validated by resequencing

| ID of VTEST | Matched gene/Accession No./% similarity/length of match (bp) | EST-SNP sequence context/position* |
|---|---|---|
| VTEST 85 | *Gallus gallus* cofilin mRNA/M55659/98% 648/661 | ccatggct(tct, S → tgt, W)ggagtaacag/49† |
| | | tttaatgac(atg, M, → ttg, L)aaagtaa/93† |
| | | aagaaag(c/g)cgttctcttctgct/145 |
| | | gagacaaagga(a/g)tctaag(aag, K → agg, R)ga/320, 328† |
| | | aggtattaaacatga(g/a)tggcaagta/443 |
| | | cagacaagt(g/a)ccatctggatcta/557 |
| | | tggaatagt(a/g)ttagtctcccttt/610 |
| | | cctggtagtttta(t/c)gtaggatccaa/734 |
| | | ggtgggatgg(t/c)agactctatac/900 |
| | | gcacacaaca(c/t)tatgcatttaa/1018 |
| | | catatctta(t/c)aaatgaagtagct/1150 |
| | | aaacatcgg(t/c)catgatggca/1270 |
| VTEST 56 | *Gallus gallus* collapsin response mediator protein/U17 277/99% 639/642 | gtgggaagatc(gtc, V → gcc, A)aatgacgac/375† |
| | | tgattac(tcc, S → tgc, C)ctgcacgtggac/708† |
| | | tggctttagc(ttg, L → atg, M)tctggcgcact/1922† |
| VTEST 137 | *Gallus gallus* elongation factor 1α mRNA/L00677/99% 643/649 | ctaaagacca(t/c)ccgaaatgggaa/55 |
| | | aggaccatcg(a/g)gaagttcg/aagaa/179,187 |
| | | cttttgtgcca(a/g)tctctggttggaacgg/637 |
| | | caactgaca(a/g)acctctgcgtct/791 |
| | | gaaagatgtccgc(cgt,R → ggt,G)ggtaacgttg/1024† |

* Each sequence context is followed by the position or locus of the SNP in the GenBank sequence of the matched *Gallus gallus* gene. Within each sequence context, the two alleles at the SNP locus are shown in parentheses. Each allele was observed in a minimum of two chromosomes or a frequency of 10% in a commercial population previously described (Smith *et al.*, 2002)
† Represents a non-synonymous change. The amino acid and codon changes are both indicated.

Copyright © 2005 John Wiley & Sons, Ltd. 

The three non-synonymous SNPs described may be useful in further defining its role in skeletal function and the dynamics of actin filaments. Similarly, the recently discovered collapsin response mediator gene product is thought to have a role in the incidence and/or severity of Alzheimer's disease (Yoshida *et al.*, 1998). The SNPs described in this gene in *Gallus gallus* may be useful in investigating the role of this apparently important gene that is also expressed in the chicken pineal gland.

It is not surprising that only 45% of the ESTs aligned to GGA1-6 DNA sequences, which comprise approximately 65% of the chicken genome. In their analysis of the draft sequence, the International Chicken Genome Sequencing Consortium (2004) reported that the density of CpG islands showed a strong negative correlation with chromosome length. This distribution supports earlier studies by McQueen *et al.* (1998) and Smith *et al.* (2000) of a higher density of genes on the microchromosomes than on the macrochromosomes. Several explanations are possible for the 9% of ESTs that did not match known sequences in GenBank, including novelty in vertebrates, too short to match known sequences, or contamination. In a recent comparative gene analysis between the chicken and human genomes, Castelo *et al.* (2005) predicted that the undiscovered genes in the human gene set may be very low, at a predicted lower limit of about 0.2%.

The number of ESTs and SNPs described in the present work are small relative to the total numbers of both genomic reagents currently available in GenBank and other databases. That they are potentially useful, however, is evident by the novelty of some of the sequences. Since a significant fraction matched mammalian genes and/or DNA sequences, they can be used as resources for comparative genome analysis of genes expressed in the pineal gland. Such comparative analysis may be useful in assigning function to chicken sequences. A similar impact on chicken biology is also likely with the SNPs described. Finally, it is worthy of note that one of the ESTs matched a W chromosome-assigned sequence. Currently, the number of genes assigned to this chromosome is limited. As efforts such as ours, even though limited in scope, identify additional ESTs, it will provide the genomic reagents essential to further increase our understanding of a chromosome that continues to be little understood.

### Acknowledgements

We thank Dr Alain Vignal of the National Institute of Agricultural Research, France, for providing the RH panel, and Kwaku Gyenai in the Comparative Genomics Laboratory at VT for the EST homology data from BLAST analysis. We are also grateful to three anonymous reviewers whose comments were used to make an extensive revision to the Results and Discussion.

### References

Arber S, Barbayannis FA, Hanser H, *et al.* 1998. Regulation of actin dynamics through phosphorylation of cofilin by LIM-kinase. *Nature* **393**: 805–809.

Bailey MJ, Beremand PD, Hammer R, *et al.* 2003. Transcriptional profiling of the chick pineal gland, a photoreceptive circadian oscillator and pacemaker. *Mol Endocrinol* **17**: 2084–2095.

Buetow KH, Edmonson MN, Cassidy AB. 1999. Reliable identification of large numbers of candidate SNPs from public EST data. *Nat Genet* **21**: 323–325.

Chong N, Bernard M, Klein DC. 2000. Characterization of the chicken serotonin N acetyltransferase gene. *J Biol Chem* **42**: 32 911–32 998.

Castelo R, Reymond A, Wyss C, *et al.* 2005. Comparative gene finding in chicken indicates that we are closing in on the set of multi-exonic widely expressed human genes. *Nucleic Acids Res* **33**: 1935–1939.

Ebihara S, Oshima I, Yamada H, Goto M, Sato K. 1987. Circadian organization in the pigeon. In *Comparative Aspects of Circadian Clocks*, Hiroshige T, Honma K (eds). Hokkaido University Press: Sapporo, Japan.

Ewing B, Hillier L, Wendl MC, Green P. 1998. Base-calling of automated sequencer traces using phred. I. Accuracy assessment. *Genome Res* **8**: 175–185.

Hubbard S, Grafhman DV, Beattie KJ, *et al.* 2005. Transcriptome analysis for the chicken based on 1626 finished cDNA sequences and 485 337 expressed sequence tags. *Genome Res* **15**: 174–183.

International Chicken Genome Sequencing Consortium. 2004. Sequence and comparative analysis of the chicken genome provide unique perspectives on vertebrate evolution. *Nature* **432**: 695–716.

International Chicken Polymorphism Map Consortium. 2004. A genetic variation map for chicken with 2.8 million single-nucleotide polymorphisms. *Nature* **432**: 717–722.

Levin I, Santangelo L, Cheng H, Crittenden LB, Dodgson JB, 1994. An autosomal genetic linkage map of the chicken. *J Hered* **85**: 79–85.

McQueen HA, Siriaco G, Bird AP, 1998. Chicken microchromosomes are hyperacetylated, early replicating, and gene rich. *Genome Res* **8**: 621–630.

Morisson M, Lemiere A, Bosc S, *et al.* 2002. ChickRH6: a chicken whole-genome radiation hybrid panel. *Genet Sel Evol* **34**: 521–533.

Okano T, Fukada Y. 2001. Photoreception and circadian clock system of the chicken pineal gland. *Microsc Res Tech* **53**: 72–80.

Okano T, Yamamoto K, Okano K, *et al.* 2001. Chicken pineal clock genes: implication of BMAL2 as a bidirectional regulator in circadian clock oscillation. *Genes Cells* **6**: 825–836.

Copyright © 2005 John Wiley & Sons, Ltd. 

Rozen S, Skaletsky HJ. 1997. Primer 3. Code available at; **http://www-genome.wi.mit.edu/genome_software/other/primer3.html**.

Semple-Rowland S, Larkin P, Bronson JD, *et al*. 1999. Characterization of the chicken GCAP gene array in analyses of GCAP1, GCAP2, and GC1 gene expression in normal and rd chicken pineal. *Mol Vision* **5**: 14.

Smith J, Bruley CK, Paton IR, *et al*. 2000. Differences in gene density on chicken macrochromosomes and microchromosomes. *Anim Genet* **31**: 96–103.

Smith EJ, Shi L, Drummond P, *et al*. 2001. Expressed sequence tags for the chicken genome from a normalized 10 day-old White Leghorn whole embryo cDNA library: 1. DNA sequence characterization and linkage analysis. *J Hered* **92**: 1–8.

Smith EJ, Shi L, Smith G. 2002. Expressed sequence tags for the chicken genome from a normalized 10-day-old white leghorn whole-embryo cDNA library. 3. DNA sequence analysis of genetic variation in commercial chicken populations. *Genome* **45**: 261–267.

Takanaka Y, Okano T, Iigo M, Fukada Y. 1988. Light-dependent expression of pineal gene in chicken pineal gland. *J Neurochem* **70**: 908–913.

Yoshida H, Watanabe A, Ihara Y. 1998. Collapsin response mediator protein-2 is associated with neurofibrillary tangles in Alzheimer's disease. *J Biol Chem* **273**: 9761–9768.

Yoshimura T, Suzuki Y, Makino E, *et al*. 2000. Molecular analysis of avian circadian clock genes. *Mol Brain Res* **78**: 207–215.

Copyright © 2005 John Wiley & Sons, Ltd. 
